# Seed Zone Nutritional Sensitivity and Hormone-Independent Rooting in Sugar Pine (*Pinus lambertiana* Dougl.): A Two-Phase Evaluation of Nutrient Solutions and Rooting Environments

**DOI:** 10.3390/plants15060981

**Published:** 2026-03-23

**Authors:** Jaime Barros Silva Filho, Arnaldo R. Ferreira, Milton E. McGiffen

**Affiliations:** 1 Contractor, Pacific Southwest Research Station, US Forest Service, United States Department of Agriculture (USDA), 4955 Canyon Crest Dr., Riverside, CA 92507, USA; 2 US Forest Service, United States Department of Agriculture (USDA), 1400 Independence Ave. SW, Yates Building, Washington, DC 20250, USA; 3 Department of Botany and Plant Sciences, University of California at Riverside, 900 University Avenue, Riverside, CA 92521, USA

**Keywords:** auxin, hydroponic system, nutrient solution, reforestation, vegetative propagation

## Abstract

Clonal propagation of rust-resistant sugar pine (*Pinus lambertiana* Dougl.) is currently limited by extreme rooting recalcitrance and highly variable donor responses to nursery management. This study identified seed zone-specific nutritional sensitivities and evaluated rooting success; we hypothesized that northern seed sources would exhibit greater sensitivity to high nutrient loads and that stable microclimates would outperform high-intensity rooting systems. In Study 1, seedlings from five United States Department of Agriculture seed zones were grown for 27 weeks in five nutrient solutions (tap-water control, modified Hoagland, Foliage-Pro^®^, Andrejow, and FloraNova^®^) spanning 0.72–3.00 dS m^−1^. The nutrient-rich Foliage-Pro^®^ and FloraNova^®^ solutions defined the upper end of the nutrient-intensity range and revealed strong seed zone contrasts: northern zones (526, 550) showed marked sensitivity, with survival declining from 70 to 100% in the control to 15–40% under the highest-EC formulations, whereas southern zones (992, 993) maintained high survival (≥75%) across all treatments and exhibited increased branching (up to 3.7 branches plant^−1^) under higher-nutrient solutions. In Study 2, stem cuttings were rooted in three environments (non-mist, hydroponic, and aeroponic) and four hormone treatments (control, Clonex^®^, Dip’n Grow^®^, and IBA + Ethrel). Rooting occurred exclusively in the non-mist propagator; untreated controls achieved 65% success and outperformed all hormone treatments (0–10%). These results demonstrate that *P. lambertiana* propagation depends on seed zone-specific donor nutrition and stable, hormone-independent rooting environments.

## 1. Introduction

Sugar pine (*Pinus lambertiana* Dougl.) is a foundational species in western North American forests [1], yet it now faces unprecedented pressure from widespread increases in tree mortality [2]. Shifting fire regimes, characterized by mass fire behavior that eludes standard operational models [3,4], have significantly reduced the probability of natural seedling survival across post-wildfire landscapes [5]. According to historical forest resource trends [6], the species is increasingly vulnerable to drought-induced bark beetle mortality [7] and the invasive pathogen white pine blister rust [8]. Ensuring future forest resilience relies on identifying individuals that persist despite these pressures [9] and preserving the adaptive diversity found within these populations [10]. Additionally, successful reforestation requires the ability to produce and move this resilient material to environments where it is most likely to thrive [11].

Clonal propagation offers a practical way to multiply resistant genotypes [12]. Despite their resilience in the wild, adult sugar pines are notoriously stubborn when it comes to rooting [13,14]. Building on general propagation guidelines for conifers [15] and recent reviews of clonal conservation methods [16], hydroponic systems emerge as a promising approach for producing juvenile stock plants. These systems allow growers to regulate nutrient availability with precision [17], creating physiological conditions that support vigorous growth without accelerating maturation [18].

The conceptual bridge between cultivation and cloning is the stock plant’s physiological readiness for adventitious rooting. Soilless cultivation alters root morphology, carbohydrate allocation, and hormonal dynamics, and these shifts influence a donor shoot’s readiness to form roots [19,20]. By manipulating nutrient regimes in hydroponic stock plants, we can modify the tissue’s internal resource status and functional readiness [21]. In this study, we used morphological parameters (survival, diameter, and growth parameters) as integrated phenotypic indicators of these underlying physiological conditions, providing a practical basis for defining a ‘nutritional window’ relevant to clonal propagation [22].

Vegetative propagation of conifers depends on rooting environments capable of supporting adventitious root formation in material that is often physiologically difficult to root. Recent syntheses highlight that successful rooting in forest trees often comes down to choosing propagation systems that match the specific physiological needs of each species [23]. The non-mist propagator (also referred to as a container-chamber rooting system) has traditionally been used for pine cuttings because it provides stable moisture and humidity conditions, typically maintaining relative humidity above 80%, which helps prevent desiccation and allows cuttings to initiate roots [24,25,26]. Hydroponic systems add another layer of control by regulating nutrient availability and oxygenation, which may support physiological conditions associated with rooting competence. While the direct rooting of conifer cuttings in hydroponic setups remains largely unexplored, these systems may contribute to physiological conditions favorable for rooting in mature clones [27,28]. Aeroponic systems deliver an oxygen-rich nutrient mist to suspended cuttings, creating conditions reported to support root initiation in other species. They have been successfully used in forestry nurseries to test root growth potential, providing aerated mist and enabling easy monitoring of root development [29]. However, their use for directly rooting temperate conifer cuttings, including *P. lambertiana*, remains largely unexplored.

While nutritional optimization provides the necessary metabolic foundation, the actual ‘switch’ that triggers root development is hormonal. Adventitious rooting is a complex developmental shift that requires a cutting to fundamentally reprogram its cellular identity, moving from stem tissue to root tissue [30,31]. This process is orchestrated by auxins, which act as the primary chemical signals for cell division and differentiation [22].

In many conifers, endogenous auxin levels naturally decline with age, contributing to the recalcitrance observed in mature trees [32,33]. To overcome this physiological barrier, exogenous application of synthetic auxins, such as indole-3-butyric acid (IBA) and 1-naphthaleneacetic acid (NAA), is commonly used to ‘jumpstart’ root primordia initiation [34]. However, the success of these treatments is not merely a matter of application; it depends on the cutting’s ability to perceive and transport these signals, a process associated with the stock plant’s physiological condition [35].

Evidence from *Pinus* spp. indicates that auxin sensitivity and transport dynamics decline with ontogenetic age and that donor-plant carbohydrate and nutrient status are associated with differences in auxin responsiveness reported in other conifers; consequently, auxin-based treatments are more likely to succeed when donor plants are physiologically juvenile or nutritionally optimized [36,37,38]. Mechanistically, changes in polar auxin transport and physiological signaling underlie this decline, indicating that nutritional status may be linked to auxin responsiveness in some conifers [39,40].

Identifying an operational nutritional window for stock plant development and evaluating how rooting environments and exogenous hormones influence adventitious rooting are essential steps in developing reliable propagation protocols for *P. lambertiana*. To address these phases, we conducted two independent studies aligned with these objectives. Study 1 assessed seed zone-specific responses to a gradient of nutrient loading intensities to identify an operational nutritional window. Study 2 evaluated whether the specific rooting environment and auxin formulations convert donor-plant quality into successful rooting. The studies were conducted independently; the stems utilized in Study 2 were harvested from a separate population of donor plants from those evaluated in Study 1.

## 2. Materials and Methods

**Figure 1:**
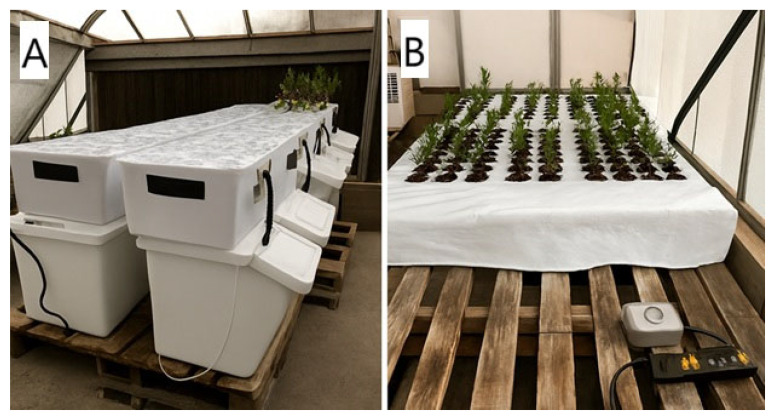
Hydroponic systems used in both studies: a small-scale ebb-and-flow setup for identifying a nutritional window (**A**, Study 1) and a flood table used for stock plant maintenance (**B**, Study 2).

We conducted two independent studies to evaluate the growth of sugar pine seedlings under a hydroponic system, using five different nutrient solutions and seedlings from five distinct seed zones. Additionally, we used three different rooting environments, with and without the addition of rooting hormones. Figure 1 illustrates the seedling growing systems used in both studies.

### 2.1. Location and Environmental Conditions

The studies were conducted in a heated glasshouse at Agricultural Operations, University of California, Riverside (Latitude 33°57′52.36″ N and Longitude 117°20′0.99″ W, 1000 feet or 305 m above sea level). Temperature and humidity were regulated by an automated evaporative cooling and heating system, with daytime and nighttime settings of approximately 25 °C and 18 °C, respectively. These values were not instrumentally measured but reflect standard operational parameters. Relative humidity was similarly maintained between 50% and 60% based on system calibration, without sensor-based logging. Photoperiod conditions followed natural seasonal variation, ranging from 10 to 11 h in October 2021 to 12–13 h in April 2022. Light intensity was not measured; however, ambient irradiance in the glasshouse typically ranges from 200 to 500 μmol·m^−2^·s^−1^ PAR, consistent with typical greenhouse conditions for temperate conifers. CO_2_ concentration was not actively controlled and remained at ambient atmospheric levels. These conditions represent typical greenhouse parameters and support the studies’ reproducibility.

### 2.2. First Study

Sugar pine stock plant seedlings were obtained from Placerville Nursery. The hydroponic system was installed on 3 October 2021 (Figure 1A).

**Figure 2:**
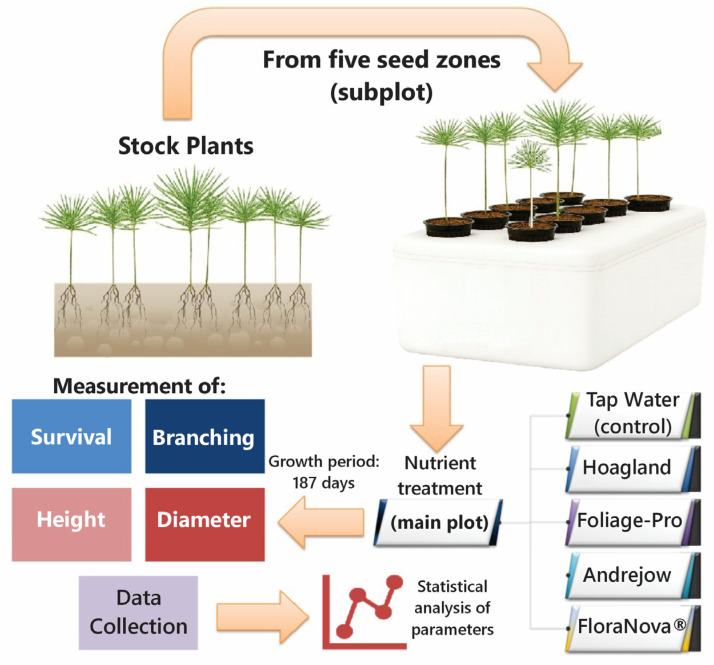
Flow diagram of the experimental process from stock plant seedlings to data analysis of survival and growth parameters.

The seedlings were placed in a RediRoot RR18 air-pruning pots filled with coconut coir growing media on an ebb-and-flow hydroponic system (Figure 2).

#### 2.2.1. Hydroponic System

The ebb-and-flow hydroponic system consisted of a 36.83 cm × 53.98 cm × 20.96 cm white polypropylene tray, with a 3.81 cm Styrofoam lid containing twenty 4.45 cm holes to accommodate RR18 air-pruning pots (RediRoot, Centralia, WA, USA). The nutrient solution reservoir consisted of a 61 L polypropylene bin (IKEA, Älmhult, Sweden). For the nutrient solution drainage system, we used 12.70 mm (½-inch) black tubing to connect the tray to the reservoir. The nutrient solution was aerated intermittently with an EcoPlus air pump (Sunlight Supply, Inc., Vancouver, WA, USA). This was connected to a 10.16 cm × 5.08 cm air stone cylinder through 4.76 mm (3/16-inch) airline tubing. To pump the nutrient solution from the 61 L reservoir to the growth tray, we used an 18.3 L min^−1^ (290 GPH) submersible pump, driven by a 7-day programmable digital outlet timer with a weekly schedule of three times per week, each lasting 2 min.

#### 2.2.2. Nutrient Solution

These five solutions were selected to comprise discrete, established laboratory and commercial nutrient formulations spanning low to high total ionic strength. This approach allowed the identification of a suitable “nutritional window” across diverse seed zones rather than generating dose–response curves for individual elements. The formulations included (1) a tap-water control [41]; (2) a modified Hoagland solution [42]; (3) Foliage-Pro^®^ 9-3-6 (representing the percentage of total nitrogen [N], available phosphate [P_2_O_5_], and soluble potash [K_2_O], respectively) [43]; (4) an Andrejow solution adapted from conifer mini-hedge protocols [44]; and (5) the nutrient-rich FloraNova^®^ Grow 7-4-10 (7% N, 4% P_2_O_5_, and 10% K_2_O) [45].

All nutrient solutions were prepared using the local municipal water supply; specifically, the commercial concentrates Foliage-Pro and FloraNova^®^ Grow were diluted to 2.6 mL L^−1^ (10 mL per gallon). The tap-water control served as an unmodified baseline (EC = 0.72 dS m^−1^); no supplemental nutrients were added to this treatment. To ensure a scientifically rigorous baseline, the chemical properties of the source water were verified by the USDA-ARS US Salinity Laboratory [41]. This characterization allowed the study to evaluate whether the inherent mineral content of the local water supply was sufficient to maintain the vigor of specific seed zones or whether it contributed to the nutritional sensitivity observed under higher-intensity regimes without the risk of nutrient toxicity. The complete chemical composition and elemental breakdown of all five nutrient solutions are detailed in Table 1. 

Initial target EC values for each solution were established at the time of preparation and are detailed in Table 1. To maintain the stability of the nutritional environment, EC and pH were monitored weekly using an HI99300 Portable Meter and HI98128 pHep^®^5 Tester (Hanna Instruments, Woonsocket, RI, USA). Solutions were adjusted to a target pH of 5.8 and fully replaced whenever the measured EC dropped by 30% from the initial concentration. This management strategy ensured that the stock plants were exposed to consistent nutrient loading intensities throughout the study period.

#### 2.2.3. Stock Plant Seedlings

Seedlings originated from five USDA seed zones spanning the Sierra Nevada and southern California: 526 (Eldorado NF), 540 (central Sierra Nevada), 550 (south-central Sierra Nevada), 992 (Los Padres NF), and 993 (San Bernardino NF) [46].

#### 2.2.4. Design

The experiment was a 5 × 5 split-plot arrangement within a randomized complete block design, with five replications, with greenhouse benches as the blocking factor. Five levels in the main plot: tap-water control, Hoagland, Foliage-Pro, Andrejow, and FloraNova^®^; and five levels in the subplot: 526, 540, 550, 992, and 993.

Each experimental unit comprised one nutrient solution and one seed zone. Each seed zone contained four seedlings as a subsample. Thus, each nutrient × seed zone treatment was represented by 4 seedlings per replication (*n* = 4) and 20 seedlings across the five replications (*n* = 20 per treatment combination). The full experiment comprised 500 seedlings in total (25 treatment combinations × 4 seedlings × 5 replications).

#### 2.2.5. Data Collection

The following parameters were evaluated for the stock plant seedlings: i. survival percentage was measured 230 days after transplanting; ii. the number of lateral branches was measured 230 days after transplanting; and iii. height growth and stem diameter were measured at the beginning (43 days after transplanting) to establish the stock plant seedlings and again after approximately 6 months (234 days after transplanting). This procedure resulted in a total growth period of 191 days. Height growth was measured at the substrate surface. Growth diameter was measured approximately 5 cm from the substrate surface.

### 2.3. Second Study

**Figure 3:**
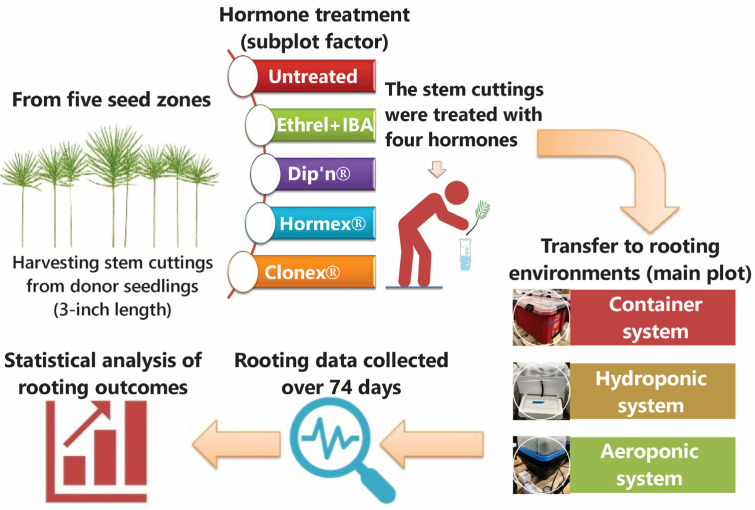
Experimental workflow for harvesting stem cuttings and assessing rooting outcomes across hormone and environment treatments.

On 3 March 2022, we harvested terminal stem cuttings from mixed-seed zone stock plant seedlings and pooled them to ensure a representative distribution of the five seed zones across treatments. Cuttings were standardized to approximately 7.5–10 cm in length and immediately assigned to receive one of four hormone treatments (IBA + Ethrel, Dip’n Grow^®^, Hormex^®^ Vitamin B1, and Clonex^®^) or remain as an untreated control. We immediately placed the cuttings into one of three rooting environments: a 76 L container (Ray Leach cells on racks filled with standard coconut coir), an ebb-and-flow hydroponic system, or an aeroponic system. We assessed rooting percentages after 74 days (Figure 3).

#### 2.3.1. Maintenance of Stock Plants

The stock plant seedlings used to supply cuttings were grown in an ebb-and-flow hydroponic system (Figure 1B) using a 1.22 m × 1.83 m Active Aqua^®^ flood table. All plants received Foliage-Pro 9-3-6 at 2.6 m L^−1^ (10 mL per gallon) to maintain uniform nutritional status. The system consisted of the flood table lined with polyethylene film and connected to a 435 L nutrient reservoir equipped with a submersible pump for irrigation and an air pump with air stones for aeration. The nutrient solution was maintained at pH 5.8 and replenished as needed. Irrigation cycles were programmed to run three times per week for 15 min. Before use, the flood table and liner were disinfected with a 15% bleach solution, and all fittings were checked to ensure proper operation.

#### 2.3.2. Nutrient Solution

The stock plants, which would later produce cuttings for the rooting study, were fed only Foliage-Pro 9-3-6. This ensured that all stock plant seedlings received the same nutrient levels. We chose to use Foliage-Pro because, in the first study, the stock plants exhibited satisfactory growth parameters across various seed zones. By using a single nutrient solution, we reduced the likelihood that differences in rooting success were attributable to initial nutrition rather than to the hormonal or environmental treatments under study. Therefore, Foliage-Pro provided a stable base and performed well in Study 1. This allowed us to focus on the fundamental question of Study 2: whether rooting hormones and environmental conditions were associated with differences in rooting success, since the mother plants were already healthy and nutritionally balanced.

Table 2 shows the nutrient concentrations recommended by Andrejow [44] for rooting cuttings.

Nutrient solution management followed the same procedures described for Study 1, with pH maintained at 5.8 and the solution replenished when EC declined by approximately 30%.

#### 2.3.3. Hormone Treatments and Composition

These four hormone treatments were selected to represent diverse auxin combinations and delivery formats. IBA + Ethrel was applied as a sequential treatment: a 10 min basal soak in 19.7 mM IBA (0.4%), followed by a 24 h soak in 69.2 µM ethephon (0.001%) to modulate auxin sensitivity, as described by Mori et al. [47], which has been reported to influence auxin-related responses in other conifers. Dip’n Grow^®^ offers dual auxin formulations diluted 5X in water. Hormex^®^ includes thiamine as a metabolic catalyst. Clonex^®^ provides a gel-based IBA treatment with supplemental vitamins and minerals for localized support. Full compositions and application methods are detailed in Table 3. Terminal stem cuttings (average length: 7.5 cm) were harvested from the mixed-seed zone stock plant seedlings. The cuttings were randomly pooled to ensure a representative mix of the five seed zones across all treatments. They were then subjected to one of five hormone treatments (Table 3) before being placed into the rooting environments.

#### 2.3.4. Rooting Environments

**Figure 4:**
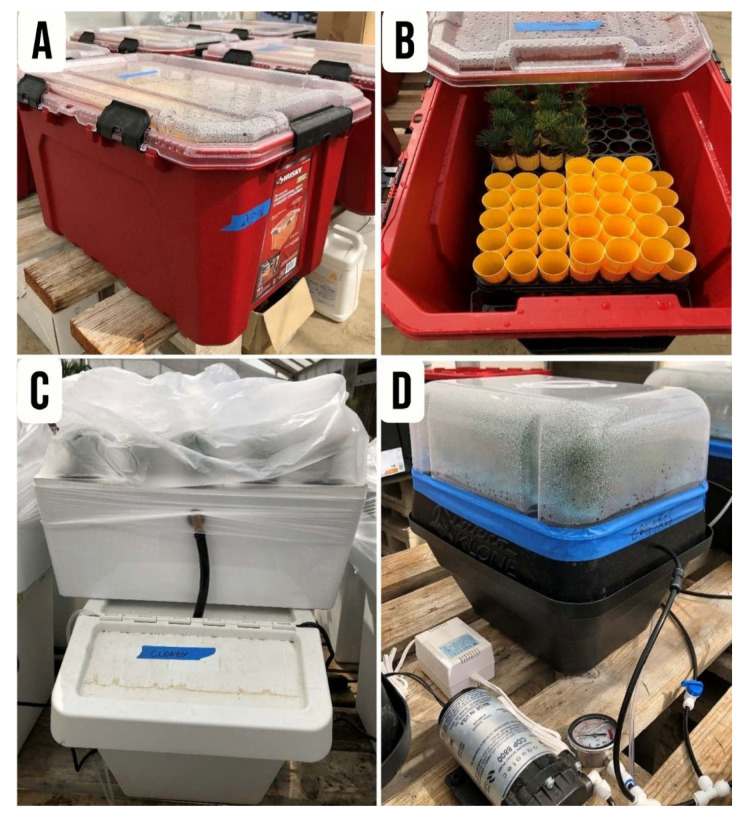
Rooting environments used in Study 2: 76 L non-mist propagator container (**A**,**B**), hydroponic system (**C**), and aeroponic system (**D**).

- A 76 L Container Rooting Environment System: Stem cuttings were placed in Ray Leach Super Cells (Stuewe & Sons, Inc., Tangent, OR, USA) filled with coconut coir. These were housed within a 76 L heavy-duty polypropylene storage container (Husky; The Home Depot, Atlanta, GA, USA). The system was sealed with a transparent lid for 74 days (Figure 4A,B);
- Hydroponic Rooting Environment System: Stem cuttings were placed in RR18 air-pruning pots filled with coconut coir. Pots were suspended through a 38.10 mm (1½-inch) Styrofoam lid fitted onto a polypropylene growth tray, as described in the first study. To maintain high humidity, the entire system was enclosed in a transparent polyethylene bag for 74 days (Figure 4C);
- Aeroponic Rooting Environment System: This system was constructed using a modified T24 Turboklone unit (Turboklone, Sacramento, CA, USA) equipped with a humidity dome. Misting was achieved via an orange DAN fogger nozzle (13.63 L h^−1^; Netafim, Tel Aviv, Israel) operated at 275.8 kPa (40 psi). The misting cycle (3 s on; 20 s off) was regulated by a digital interval timer (Nearpow, Shenzhen, China). System pressure was maintained using an 8800 Booster Pump (Aquatec, Irvine, CA, USA). Stem cuttings were secured with foam collars, and the junction between the unit base and the humidity dome was sealed with adhesive tape for 74 days (Figure 4D). The nutrient solution was refreshed weekly with tap water; the pH was adjusted to 6.0, and the solution was replenished when the electrical conductivity (EC) decreased by 30% from the initial concentration.

#### 2.3.5. Design

The experiment was a 3 × 5 factorial arrangement within a completely randomized design with two replications. Three levels of rooting environments (76 L container, hydroponic system, and aeroponic system) were combined with five levels of hormone treatments: an untreated control, IBA plus Ethrel, Dip’n, Hormex^®^ Vit. B1, and Clonex^®^ containing 0.31% IBA. To minimize positional bias, treatment combinations were randomly assigned to glasshouse benches. Each hormone × environment treatment combination was replicated twice, with 10 cuttings per replication (i.e., *n* = 20 cuttings per hormone × environment treatment). With 15 treatment combinations (3 environments × 5 hormone levels), the experiment included 300 cuttings in total.

#### 2.3.6. Data Collection

Rooting percentage was selected as the primary response variable because many cuttings produced only early-stage root initials at the 74-day harvest, and these fragile structures could not be measured reliably without risk of breakage. Quantifying root number, length, or quality would have introduced substantial measurement bias. As rooting protocols for *P. lambertiana* improve and more robust root systems develop, future studies should incorporate detailed root morphological traits to complement rooting success.

#### 2.3.7. Data Analysis (Applies to Both Studies)

For Study 1, seed zone and nutrient solution effects were analyzed using a two-way ANOVA within a split-plot design. The nutrient solution served as the main plot factor (five levels), and the seed zone served as the subplot factor (five levels), with blocks treated as a random effect. For Study 2, rooting environment and hormone treatment were analyzed as fixed factors in a two-way factorial design. Prior to analysis, all data were evaluated for normality and homogeneity of variance, and means were compared using Tukey’s HSD test (*p* < 0.05).

To quantify the magnitude of treatment effects in Study 1, partial eta-squared ($\eta_{p}^{2}$) values were calculated following established procedures for hierarchical designs [48,49,50]. Because of the split-plot structure, $\eta_{p}^{2}$ was computed separately for each stratum. For the main plot factor (nutrient solution), $\eta_{p}^{2}$ was calculated as follows:

$$\eta_{p}^{2}=\frac{{SS}_{NS}}{{SS}_{NS}+{SS}_{Error\;A}}$$

For subplot factors (seed zone and the seed zone × nutrient solution interaction), $\eta_{p}^{2}$ was calculated as follows:

$$\eta_{p}^{2}=\frac{{SS}_{factor}}{{SS}_{factor}+{SS}_{Error\;B}}$$

where SS denotes the sum of squares, and NS denotes the nutrient solution. These effect-size values provide a standardized measure of the variance explained by each factor after accounting for the nested error structure of the split-plot design. All analyses were performed in SAEG 9.1 [51], and graphs were generated in OriginPro 2025 [52].

## 3. Results

### 3.1. First Study

Survival patterns varied markedly across the nutrient solution × seed zone combinations. In northern zones 526 and 550, unmodified tap-water control supported the highest survival (70.00 ± 12.25% and 100.00 ± 0.00%, respectively), while enriched formulations such as Foliage-Pro and FloraNova^®^ reduced survival to 15.00 ± 6.12% and 30.00 ± 14.58%, respectively. In contrast, southern zones 992 and 993 maintained high survival across all treatments, with values ranging from 65.00 ± 10.00% to 100.00 ± 0.00%. Zone 540 showed improved survival under tap-water control (90.00 ± 10.00%) compared to nutrient-enriched treatments (Table 4).

Lateral branching also varied across seed zones and nutrient solutions. Seedlings from Zone 992 exhibited the highest branching under Foliage-Pro (3.65 ± 1.24), FloraNova^®^ (3.40 ± 1.13), and Andrejow (3.25 ± 0.90). Zone 993 showed elevated branching under Hoagland (2.75 ± 0.38) and Foliage-Pro (2.60 ± 0.74). In contrast, zones 526 and 550 maintained low branching across all treatments, with values ranging from 1.15 ± 0.13 to 2.40 ± 0.19. Zone 540 showed moderate branching under Foliage-Pro (2.45 ± 0.46) and Andrejow (2.25 ± 0.44), as quantified in Table 4.

Seedling height growth varied across seed zones and nutrient solutions, with southern zones generally exhibiting greater responsiveness to nutrient enrichment. In seed zone 992, Andrejow produced the highest mean height (2.33 ± 0.53 cm), followed by FloraNova^®^ (1.53 ± 0.28 cm) and Foliage-Pro (1.65 ± 0.44 cm). Seed zone 993 also responded strongly to Andrejow (2.08 ± 0.47 cm), with moderate growth under Foliage-Pro (1.43 ± 0.49 cm) and Hoagland (1.30 ± 0.30 cm). In contrast, northern zones 526 and 550 showed minimal variation across treatments, with height growth remaining below 1.00 cm in all cases (Table 5).

Stem diameter followed a similar pattern. Seed zone 993 achieved the highest diameter under Andrejow (1.12 ± 0.13 mm) and Hoagland (1.07 ± 0.07 mm), while zone 992 reached 0.91 ± 0.18 mm under Foliage-Pro. Northern zones 526 and 550 exhibited lower stem diameters across all treatments, with values ranging from 0.30 ± 0.07 mm to 0.54 ± 0.21 mm. These patterns are quantified in Table 5.

Partial eta-squared ($\eta_{p}^{2}$) values quantify the substantial impact of the nutrient solution × seed zone interaction. The interaction effect was most pronounced for survival ($\eta_{p}^{2}$ = 0.31) and height ($\eta_{p}^{2}$ = 0.25), indicating that the response to nutrition is highly dependent on genetic origin. While the nutrient solution was the dominant driver for survival ($\eta_{p}^{2}$ = 0.64), the seed zone itself played a more secondary but still significant role in traits like branching ($\eta_{p}^{2}$ = 0.20). These values confirm that both main factors and their interaction contributed meaningfully to the observed variation (Table 6).

### 3.2. Second Study

Rooting success was associated with a highly significant interaction between rooting environment and hormone treatment (F_8,15_ = 137.93; *p* < 0.0001), with the model explaining 99.3% of the observed variance (R^2^ = 0.99). Root initiation occurred only in the non-mist propagator container system; in the hydroponic and aeroponic systems, no rooted cuttings were produced under the tested conditions.

**Figure 5:**
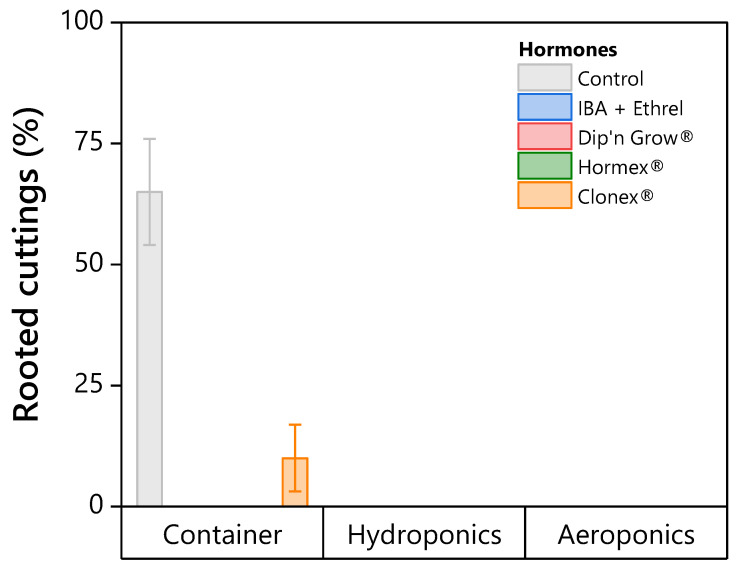
Rooting percentages by hormone treatment within each rooting environment. Bars represent mean ± standard error. Hormone treatments shown in the legend but not visible in the plot yielded 0% rooting success across all environments. For numerical values and statistical significance of zero-result treatments, see Table 7.

**Figure 6:**
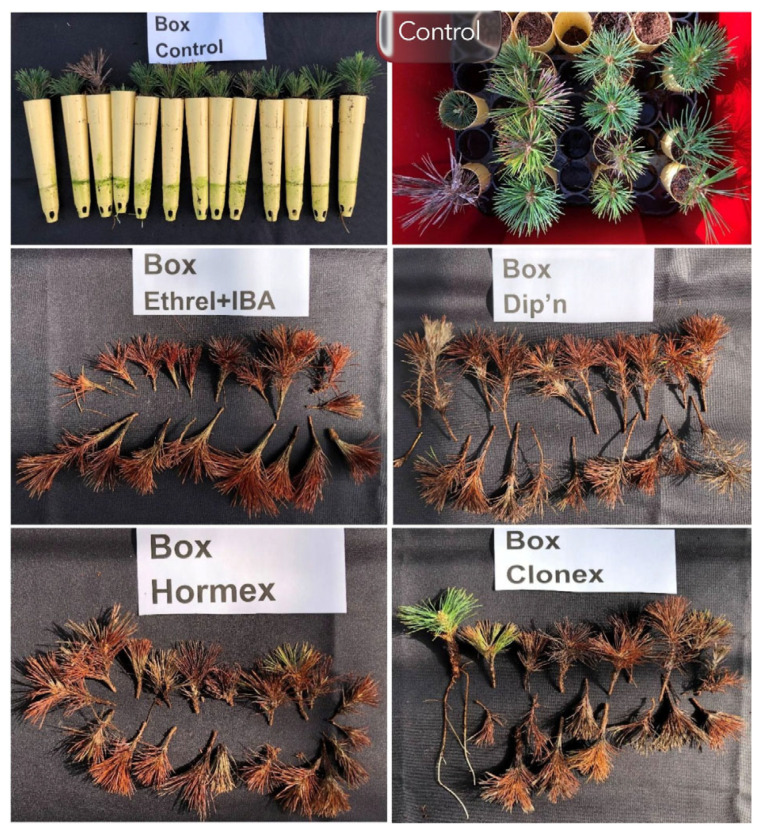
Rooted cuttings of sugar pine at 74 days after transplanting in a 76 L non-mist propagator container system (Box).

Within the non-mist propagator container system, the untreated control achieved the highest rooting percentage (65.0 ± 5.0%), and Clonex^®^ produced a lower rooting percentage (10.0 ± 2.0%). IBA + Ethrel, Dip’n Grow^®^, and Hormex^®^ showed no rooted cuttings under the tested conditions. Across environments, none of the tested hormone formulations increased rooting percentages relative to the untreated control. The untreated control consistently ranked among the highest treatments across rooting systems (Figure 5 and Figure 6; Table 7).

## 4. Discussion

### 4.1. First Study

The nutritional status of stock plants used for sugar pine vegetative propagation is critical for generating clones with resistance to white pine blister rust, wildfires, and bark beetles. Our results reveal variability in survival, growth, and branching responses across seed zones, underscoring notable genotype × nutrient interactions. Survival, lateral branching, height growth, and stem diameter were all influenced by the interaction between the seed zone and the nutrient solution, underscoring the need to integrate genetic origins with external nutritional management in conifer propagation systems. These findings are consistent with broader evidence of genotype-by-environment interactions in forest tree propagation [53,54,55]. Our results demonstrate a distinct genotype-by-environment interaction, indicating that the optimal nutrient regime for robust growth (encompassing branching, height, diameter, and survival) varies according to the specific seed zones of the stock plants.

Seedlings originating from seed zones 550 and 526 exhibited reduced nutritional requirements compared to those from seed zones 992, 993, and 540, leading to increased survival rates when provided only with a tap-water nutrient solution without added salts. This finding indicates that genotypes from these seed zones have adapted to low-fertility conditions, consistent with the concept of local adaptation to edaphic factors [56,57]. The reduced survival of these same genotypes under enriched nutrient solutions supports the possibility that excessive nutrient loading may induce osmotic or ionic stress [58,59]. Additionally, although not statistically significant, some nutrient solutions achieved maximum survival rates in seed zones 992 and 993. Differences in nutrient formulation, such as ionic balance, buffering capacity, and macro-/micronutrient ratios, may contribute to these patterns [60,61]. The relatively strong performance of Andrejow and Foliage-Pro in some seed zones suggests that their nutrient profiles more closely match the physiological requirements of certain genotypes. These results reinforce the importance of tailoring nutrient regimes to provenance rather than applying uniform fertilization protocols.

Seed Zone 540 also showed its highest survival under unmodified tap-water control, similar to northern zones, but exhibited moderate branching responses to nutrient enrichment. However, when comparing tap-water control results across all zones, no statistically significant differences emerged, indicating that low-nutrient conditions do not inherently disadvantage any genotype but disproportionately benefit those adapted to nutrient-poor environments.

The diverse responses correspond with earlier research indicating that the origin of a seedling significantly affects its vigor, stress tolerance, and nutrient absorption efficiency [62,63]. Seedlings from Seed Zones 992 and 993 exhibited elevated survival rates in diverse nutrient solutions. This suggests enhanced physiological adaptability, plasticity, and the ability to tolerate a wider range of nutrient environments, enabling the management of a broader spectrum of nutritional formulas. Such adaptability may enhance their performance in variable nursery or field conditions, a trait advantageous for large-scale propagation and restoration programs [53,64].

Our results indicate that a single nutrient plan for all seed zones is not effective. Reforestation initiatives in zones 550 and 526 are likely to be most effective with low-input, low-fertility protocols, whereas zones 992 and 993 necessitate higher-nutrient regimes. This seed zone-specific approach aligns with strategies for managing plant movement and adaptation across heterogeneous soil environments [65].

Lateral branches after clipping the pine stem cuttings are a critical trait for vegetative propagation because they determine the number of cuttings that can be harvested from each stock plant [66,67]. In seed zone 992, Foliage-Pro and Andrejow increased branching by approximately 170% and 140%, respectively, relative to tap-water control. In seed zones 993 and 540, Foliage-Pro increased branching by 189% and 123%, respectively. Although these increases were numerically substantial, only zone 992 showed statistically significant differences among nutrient treatments, as indicated by Tukey’s HSD test. Our findings at seed zones 550 and 526 indicate that nutrient solutions did not significantly influence branching. This may suggest that genotypes from these seed zones possess inherently conservative developmental strategies with limited plasticity in shoot architecture [68]. Such genotypes may be evolutionarily adapted to low-nutrient environments and therefore less responsive to nitrogen-rich formulations such as Foliage-Pro.

Conversely, seed zones 992 and 993 showed strong branching responses to nutrient enrichment. In seed zone 992, Foliage-Pro and FloraNova^®^ markedly enhanced branching relative to tap-water control and Hoagland’s solution, whereas in zone 993, Hoagland and Foliage-Pro produced numerically higher branching than tap-water control, although these differences were not statistically significant. This pattern may still reflect a degree of physiological responsiveness to nutrient enrichment, particularly in formulations with balanced macro- and micronutrient availability. Enhanced branching under nutrient-rich conditions aligns with the known roles of nitrogen, phosphorus, and carbohydrate availability in promoting axillary bud activation and shoot proliferation [69,70,71]. Our results corroborate earlier research indicating that nutrient composition may influence shoot architecture through hormonal dynamics, including cytokinins and auxins, which are known to govern axillary bud activation in other systems [72,73,74,75].

Comparisons among seed zones revealed that branching responses to nutrient solutions were not uniform. Branching is essential for vegetative propagation, influencing the availability of cuttings, canopy architecture, and plant morphology in the field. The findings demonstrate that using nutrient solutions such as Foliage-Pro or FloraNova^®^ promotes branching in the sensitive seed zones (e.g., 992), thereby improving propagation efficiency. On the other hand, in areas with low responsiveness (e.g., 550 and 526), nutrient solutions may not be worth the additional cost, and soils with low fertility (e.g., unmodified tap water) may be enough.

The results of the partial eta-squared ($\eta_{p}^{2}$) indicate a significant interaction between genetic origin and environmental resource availability in the early establishment of sugar pine (*Pinus lambertiana* Dougl.). We observed a distinct split in what drives plant development: survival is largely determined by the seed’s origin (genetics), while growth traits (like height and stem thickness) depend more on how well the plant is fed (environment). This difference shows “phenotypic plasticity,” which means that plants can change their physical appearance based on their surroundings. Consequently, these findings demonstrate the importance of restoration efforts to account for the interaction between a plant’s genetics (G) and its environment (E).

The most striking finding was the dominance of genetics in survival ($\eta_{p}^{2}$ = 0.53). In other words, 53% of the difference in survival rates came down to origin. This aligns with foundational research showing that conifers are highly adapted to their local microclimates [76,77]. Essentially, if you plant a seed outside its optimal zone (“maladaptation”), it faces risks that no amount of fertilizer or care can compensate for.

However, the story becomes more complex. We also found a strong “interaction effect” for survival ($\eta_{p}^{2}$ = 0.31). This suggests that a seedling’s survival is not determined solely by genetics; it also depends on the nutrient regime. This supports the “reaction norm” framework: different genotypes have unique response curves [78]. A seed source that struggles in low-nutrient soil might thrive if given better food, while a tough, resilient genotype might not care about the extra resources at all [79,80].

In contrast to survival, growth traits were much more flexible. The nutrient solution was the number one predictor of how tall and thick the seedlings grew. These data demonstrate that these seedlings are programmed to rapidly exploit favorable conditions—a crucial survival trait for establishing themselves in forest gaps [81]. The interaction effect for stem diameter ($\eta_{p}^{2}$ = 0.27) indicates that genotypes differ in their biomass allocation. Although improved nutrition promotes growth across all seedlings, allometric scaling (e.g., diameter-to-height ratio) appears genetically constrained, consistent with seed zone trials [82].

These results require the evaluation of individualized propagation approaches that incorporate genetic origin and nutritional solution composition. In addition, the substantial interaction effect indicates that survival outcomes cannot be optimized by nutrient solution alone; they must be considered in the context of seed zone origin. These findings suggest that restoration approaches must align with both the genotype and the environment.

Branching exhibited the smallest effect sizes ($\eta_{p}^{2}$ = 0.18–0.35), suggesting that canopy architecture may be associated with developmental constraints or environmental factors not addressed here, such as light quality, internal hormones, or photoperiod [83,84,85]. The reduced variance explained suggests that nutrient regimes are less effective at altering branching than at altering biomass accumulation.

Standardized nursery procedures are less successful when genotype-by-environment interactions are present. Reforestation success depends on matching nutrient regimes to specific genetic families, in addition to selecting fast-growing species or efficient fertilizers. Management strategies must emphasize establishing strict seed transfer zones, as the seed zone significantly influences survival, thereby helping prevent outbreeding depression and maladaptation [86]. Strategies for post-fire or nutrient-altered soil-assisted migration should account for genotype performance under new edaphic conditions, particularly by evaluating how different genetic families respond to varying nutrient regimes and soil types to ensure successful establishment and growth in altered environments [87]. Future propagation efforts should transition away from uniform fertilizer inputs to customized regimes that improve the survival of regionally adapted seed batches. The quality of the donor stock plants established in Study 1 directly influenced the potential success of the propagation protocols evaluated in the subsequent rooting trial (Study 2).

### 4.2. Second Study

Our results with rooted sugar pine cuttings demonstrated a distinct sensitivity to exogenous hormones. This finding differs from preliminary studies suggesting auxin-based rooting treatments enhance adventitious root development in pine species, yet a similar lack of positive response has been reported in loblolly and Virginia pine, particularly at elevated auxin concentrations [37,88]. Untreated cuttings showed a higher rooting percentage, consistent with reports that donor material predisposition strongly influences rooting success [35,89]. Previous and current studies on various pine species have demonstrated variable responses to hormone treatment, with specific investigations observing reduced rooting at higher dosages [35,37,89].

Hormone concentrations were not measured in this study; however, they are similar to patterns reported in other conifer species, particularly in auxin–ethylene interactions, which help interpret the rooting patterns we observed. In these systems, elevated auxin can stimulate ACC synthase activity, leading to ethylene accumulation that limits root initiation [90,91,92]. Our study, which indicated that the combination of IBA and Ethrel resulted in lower rooting percentages, indicated that this formulation may have affected hormonal dynamics under the tested conditions, echoing prior findings that exogenous ethylene can interfere with auxin-mediated root initiation in conifers [37,88,89,93].

This sensitivity differs from commercial procedures for *Pinus radiata* D. Don, which often use large doses of IBA to increase rooting percentage and speed, even in juvenile specimens [94,95]. Similarly, *Pseudotsuga menziesii* (Mirb.) Franco (Douglas-fir) often requires auxin application to mitigate dormancy- or maturation-related recalcitrance [34]. However, our results support earlier findings, indicating that some pine species may show limited benefit from external hormone applications under optimal containerized conditions [96]. For instance, the authors cite Greenwood and Goldsmith [97], who found that the phytotropin triiodobenzoic acid (TIBA) reduced rooting in sugar pine cuttings.

The results for untreated *P. lambertiana* Dougl. cuttings suggest that excellent donor nutrition (as established in Study 1) may reduce the need for exogenous auxin under certain physiological conditions, potentially rendering exogenous application unnecessary or even detrimental. Our findings demonstrate that, for vigorous stock, the optimal strategy shifts from hormone application to maximizing stock plant physiological quality and selecting the optimal rooting environment. Cuttings without hormones exhibited superior rooting in the 76 L container rooting environment system. Therefore, for *Pinus lambertiana* Dougl., standard commercial rooting hormone powders did not improve rooting success and may be unnecessary when donor plants are well managed.

Our results suggest that rooting hormones may not be universally required for conifer propagation under optimized conditions. Some nursery companies use hormone treatments to help stock plants grow more quickly. Additionally, our findings indicate that optimizing upstream donor nutrition, particularly through seed zone-specific hydroponic inputs, may eliminate the requirement for downstream auxin treatments. In the non-mist propagator system, the untreated approach yielded the highest rooting success, suggesting that internal physiological priming is a more critical determinant than exogenous chemical intervention. This shift toward stock plant management over hormonal triggers offers significant operational efficiencies by reducing labor, chemical costs, and potential phytotoxicity. This approach implies a shift toward effective management practices with stock plants, in which stock plant physiological priming may be more critical than hormone application during peg-cutting rooting.

These findings underscore the importance of optimizing stock plant management and rooting environments rather than relying on exogenous hormone treatments. This technique can be decisive for rooting success. Rooting percentage was utilized as the primary metric because many cuttings exhibited emergent adventitious root initials at the 74-day harvest. These primordial structures were highly susceptible to mechanical breakage during cleaning; consequently, to prioritize data accuracy over secondary morphological quantification, we focused on the binary success of root initiation as the most reliable biological indicator. While this approach provided a consistent and biologically meaningful indicator of treatment effects, it also represents a limitation of the study. Root number, length, and quality were not quantified, and hormone concentrations were not measured directly. In addition, donor seedlings were pooled across seed zones in Study 2, which may have masked zone-specific rooting responses. As propagation protocols for *P. lambertiana* improve and root systems become more robust, future studies should incorporate detailed root morphological traits, such as total root length, branching density, and fine-root development, along with hormone quantification and genotype-specific analyses to refine clonal propagation strategies and better predict post-transplant vigor.

Additionally, this study was designed as an applied evaluation of whole-formulation nutrient regimes and standardized rooting products under operational propagation conditions. It was not intended to partition electrical conductivity from specific ion effects or to establish auxin dose–response curves for *P. lambertiana*. Such mechanistic experiments would require controlled EC gradients and explicit auxin concentration series and are valuable next steps. Our conclusions are therefore limited to the comparative performance of the tested formulations and the observed seed zone response patterns; we encourage targeted follow-up studies to resolve EC versus element-specific effects and to identify optimal auxin dosages for this species.

By establishing these baseline nutritional windows and rooting protocols, this study provides the necessary prerequisite for subsequent investigations into the fine-scale physiological mechanisms of sugar pine propagation.

## 5. Conclusions

This research identified the nutritional and environmental conditions required for the clonal propagation of *Pinus lambertiana*. Study 1 demonstrated that donor-plant survival, diameter, and growth are significantly influenced by both nutrient solution and seed zone origin. In Study 2, high rooting percentages were achieved with a non-mist propagation system, in which the application of exogenous auxin formulations did not significantly increase rooting success. Collectively, these results define the baseline nutritional and environmental parameters necessary for the production of sugar pine clones.

## Figures and Tables

**Table 1:** Composition of five nutrient solutions used for hydroponic cultivation of sugar pine stock plant seedlings.

Nutrients	◊ Tap Water (Control)	† Hoagland	‡ Foliage-Pro	⁑ Andrejow	⁕ FloraNova^®^
	mmol L^−1^	mmol_c_ L^−1^	%	mmol L^−1^	%
Nitrate	0.42	14.00	6.10	10.70	6.10
Ammonium	0.00	1.00	2.90	1.20	0.90
Phosphorus	0.00	1.00	3.00	1.00	4.00
Potassium	0.11	6.00	6.00	4.00	10.00
Calcium	3.09	4.00	2.00	1.00	4.00
Magnesium	0.78	2.00	0.50	1.00	1.50
Sulfur	1.28	2.00	–	–	2.00
Sodium	1.70	–	–	6.50	–
	µmol L^−1^	µmol L^−1^		µmol L^−1^	
Iron	0.041	90.00 *	0.10 *	17.55 *	0.10 *
Manganese	0.000	12.60	0.05 *	16.38	0.030 *
Cobalt	0.004		0.0005	–	0.002
Boron	0.123	46.00	–	18.50	0.010
Zinc	0.035	1.30	0.05 *	5.81	0.020 *
Copper	0.097	0.30	0.05 *	1.57	0.010 *
Molybdenum	0.003	0.10	0.0009	–	0.003
**EC** (dS m^−1^)	0.72	2.60	2.48	2.27	3.00

◊ Values for tap water represent the baseline mineral content of the local supply; no supplemental salts or nutrients were added to this treatment; † NH_4_H_2_PO_4_; MnSO_4_·H_2_O; Ca(NO_3_)_2_ 4H_2_O; KNO_3_; CuSO_4_·5H_2_O; ZnSO_4_.7H_2_O; MnSO_4_·H_2_O; H_3_BO_3_; (NH_4_)_6_Mo_7_O_24_·4H_2_O; FeCl_3_·6H_2_O and C_10_H_14_N_2_O_8_Na_2_·2H_2_O (EDTA, Ethylenediaminetetraacetic Acid Disodium); ‡ ammonium nitrate, calcium nitrate, potassium nitrate, monoammonium phosphate, monopotassium phosphate, cobalt sulfate, magnesium sulfate, molybdic oxide, copper EDTA, iron EDTA, manganese EDTA, and zinc EDTA; ⁑ NH_4_H_2_PO_4_; NH_4_NO_3_; KNO_3_; NaNO_3_; CuSO_4_; ZnSO_4_.7H_2_O; MnSO_4_.H_2_O; H_3_BO_3_; FeCl_3_.6H_2_O and C_10_H_14_N_2_O_8_Na_2_.2H_2_O (EDTA); and ⁕ ammonium molybdate, ammonium phosphate, calcium nitrate, copper EDTA, iron DTPA, magnesium sulfate, manganese EDTA, monopotassium phosphate, potassium chloride, potassium citrate, potassium nitrate, sodium borate, and zinc EDTA. * Indicates nutrients provided in chelated form.

**Table 2:** Nutrient solution composition used for rooting *P. lambertiana* cuttings in Study 2. Formulations were prepared following Andrejow [44].

Nutrients	mmol_c_ L^−1^
Nitrate	5.80
Ammonium	0.60
Phosphorus	0.60
Potassium	3.00
Calcium	1.60
Magnesium	1.40
Sulfur	1.40
	µmol L^−1^
Iron (Chelated)	19.70
Manganese	34.60
Boron	27.75
Zinc	7.65
Copper	1.57

N as NH_4_H_2_PO_4_, KNO_3_, Ca(NO_3_)_2_, and NaNO_3_; P as NH_4_H_2_PO_4_; K as KNO_3_; Ca as Ca(NO_3_)_2_; Mg and S as MgSO_4_.

**Table 3:** Hormone treatments applied to sugar pine stem cuttings in Study 2.

Treatment	Active Ingredient(s)	Concentration	Form/Additional Components
Control	None	0%	Untreated baseline
IBA + Ethrel ^1^	IBA + Ethephon	0.40% + 0.001%	Liquid (10 min/24 h sequential soak)
Dip’n^® 2^	IBA + NAA	1.0% + 0.5%	Liquid (Dual-auxin, 5× dilution)
Hormex^®^ Vit. B1 ^3^	NAA + Vitamin B1	0.24% + 0.25%	Liquid (with Thiamine metabolic catalyst)
Clonex^® 4^	IBA	0.31%	Gel (with Vitamins and Minerals)

^1^ Protocol developed by Mori et al. [41], ^2^ https://dipngrow.com/ (accessed on 18 January 2026), ^3^ https://hormex.com/products/vitamin-b1-hormone-concentrate?srsltid=AfmBOordtLsBATSqQCndmcV_r-ijL6ImVzR1ssfBifamMLb2z2MdpXWc (accessed on 18 January 2026), ^4^ https://myclonex.com/ (accessed on 18 January 2026).

**Table 4:** Stock plant parameters by seed zone and nutrient solution.

Seed Zone	Nutrient Solution				
	Tap Water (Control)	Hoagland	Foliage-Pro	Andrejow	FloraNova^®^
	**Survival (%)**				
526 (North)	70.00 ± 12.25 aA	50.00 ± 7.91 aAB	15.00 ± 6.12 bB	50.00 ± 13.69 cAB	40.00 ± 20.31 bAB
540 (Central)	90.00 ± 10.00 aA	50.00 ± 7.91 aB	65.00 ± 6.12 aAB	65.00 ± 12.75 abcAB	60.00 ± 12.75 abAB
550 (North)	100.00 ± 0.00 aA	50.00 ± 17.68 aB	25.00 ± 19.36 bB	60.00 ± 6.12 bcB	30.00 ± 14.58 bB
992 (South)	100.00 ± 0.00 aA	75.00 ± 11.18 aA	90.00 ± 6.12 aA	100.00 ± 0.00 aA	80.00 ± 12.25 aA
993 (South)	95.00 ± 5.00 aA	85.00 ± 10.00 aA	100.00 ± 0.00 aA	90.00 ± 10.00 abA	65.00 ± 10.00 abA
	**Branching**				
526 (North)	1.15 ± 0.13 aA	1.30 ± 0.19 aA	1.40 ± 0.22 bA	1.90 ± 0.39 aA	1.45 ± 0.32 aA
540 (Central)	1.10 ± 0.19 aA	1.85 ± 0.29 aA	2.45 ± 0.46 abA	2.25 ± 0.44 aA	1.65 ± 0.29 aA
550 (North)	1.55 ± 0.18 aA	1.50 ± 0.11 aA	2.40 ± 0.19 abA	1.60 ± 0.23 aA	2.20 ± 0.31 aA
992 (South)	1.35 ± 0.41 aB	1.95 ± 0.60 aAB	3.65 ± 1.24 aA	3.25 ± 0.90 aAB	3.40 ± 1.13 aAB
993 (South)	0.90 ± 0.30 aA	2.75 ± 0.38 aA	2.60 ± 0.74 abA	1.80 ± 0.35 aA	1.70 ± 0.50 aA

Values represent mean ± SE (standard error). Sample size per cell = 4 seedlings per replication; five replications (*n* = 20 per treatment combination). Different lowercase letters indicate significant differences between seed zones within each nutrient solution, while uppercase letters indicate differences between nutrient solutions within each seed zone, according to Tukey’s HSD test (*p* < 0.05).

**Table 5:** Stock plant growth parameters by seed zone and nutrient solution.

Seed Zone	Nutrient Solution				
	Tap Water (Control)	Hoagland	Foliage-Pro	Andrejow	FloraNova^®^
	**Height Growth (cm)**				
526 (North)	0.83 ± 0.12 aA	0.78 ± 0.13 aA	0.80 ± 0.16 aA	0.95 ± 0.21 cA	0.90 ± 0.19 aA
540 (Central)	0.85 ± 0.27 aA	0.85 ± 0.14 aA	1.33 ± 0.36 aA	1.15 ± 0.17 bcA	0.73 ± 0.09 aA
550 (North)	0.85 ± 0.14 aA	0.60 ± 0.12 aA	1.20 ± 0.15 aA	0.88 ± 0.21 cA	0.58 ± 0.15 aA
992 (South)	0.73 ± 0.20 aB	0.95 ± 0.16 aB	1.65 ± 0.44 aAB	2.33 ± 0.53 aA	1.53 ± 0.28 aAB
993 (South)	0.75 ± 0.19 aB	1.30 ± 0.30 aAB	1.43 ± 0.49 aAB	2.08 ± 0.47 abA	1.13 ± 0.18 aAB
	**Stem Diameter (mm)**				
526 (North)	0.43 ± 0.09 aA	0.54 ± 0.21 bA	0.43 ± 0.03 abA	0.30 ± 0.07 bA	0.39 ± 0.13 aA
540 (Central)	0.12 ± 0.04 aB	0.43 ± 0.09 bAB	0.69 ± 0.14 abA	0.58 ± 0.06 bAB	0.43 ± 0.07 aAB
550 (North)	0.28 ± 0.11 aA	0.38 ± 0.07 bA	0.33 ± 0.09 bA	0.40 ± 0.05 bA	0.37 ± 0.12 aA
992 (South)	0.22 ± 0.08 aB	0.82 ± 0.18 abA	0.91 ± 0.18 aA	0.75 ± 0.13 abA	0.75 ± 0.09 aA
993 (South)	0.43 ± 0.20 aB	1.07 ± 0.07 aA	0.77 ± 0.17 abAB	1.12 ± 0.13 aA	0.74 ± 0.14 aAB

Values represent mean ± SE (standard error). Sample size per cell = 4 seedlings per replication; five replications (*n* = 20 per treatment combination). Different lowercase letters indicate significant differences between seed zones within each nutrient solution, while uppercase letters indicate differences between nutrient solutions within each seed zone, according to Tukey’s HSD test (*p* < 0.05).

**Table 6:** Partial eta squared ($\eta_{p}^{2}$) values from ANOVA quantify the effect sizes of nutrient solution, seed zone, and their interaction on survival and three growth traits in sugar pine. Higher values indicate greater variance explained by each factor.

Factors	Survival	Branching	Height Growth	Stem Diameter
$\mathrm{Nutrient}\;\mathrm{solution}\;\eta_{p}^{2}$	0.64	0.35	0.40	0.57
$\mathrm{Seed}\;\mathrm{zones}\;\eta_{p}^{2}$	0.53	0.20	0.27	0.43
$\mathrm{Interaction}\;\eta_{p}^{2}$	0.31	0.18	0.25	0.27

**Table 7:** Rooting percentages by hormone treatment and rooting environment.

Hormone Treatment	Rooting Environment		
	Container	Hydroponic	Aeroponic
Control (Untreated)	65.0 ± 5.0 aA	0.0 ± 0.0 aB	0.0 ± 0.0 aB
IBA + Ethrel	0.0 ± 0.0 cA	0.0 ± 0.0 aA	0.0 ± 0.0 aA
Dip’n Grow^®^	0.0 ± 0.0 cA	0.0 ± 0.0 aA	0.0 ± 0.0 aA
Hormex^®^	0.0 ± 0.0 cA	0.0 ± 0.0 aA	0.0 ± 0.0 aA
Clonex^®^	10.0 ± 2.0 bA	0.0 ± 0.0 aB	0.0 ± 0.0 aB

Values represent mean ± SE (standard error); *n* = 10 per replication; two replications (*n* = 20 per hormone × environment combination). Lowercase letters indicate significant differences between hormone treatments within each rooting environment; uppercase letters indicate significant differences between rooting environments within each hormone treatment (Tukey’s HSD, *p* < 0.05). No rooted cuttings were observed in hydroponic and aeroponic systems under the tested conditions.

## Data Availability

The new data generated here were created solely for testing our treatments and hypotheses and are not intended for entry into any public database or archive. Thus, data sharing does not apply to this article.
